# Preoperative prediction for early recurrence of hepatocellular carcinoma using machine learning-based radiomics

**DOI:** 10.3389/fonc.2024.1346124

**Published:** 2024-03-15

**Authors:** Bing Mao, Yajun Ren, Xuan Yu, Xinliang Liang, Xiangming Ding

**Affiliations:** ^1^ Henan Provincial People’s Hospital, Zhengzhou University People’s Hospital; Henan University People’s Hospital, Zhengzhou, Henan, China; ^2^ Department of Gastroenterology, The First Affiliated Hospital of Zhengzhou University, Zhengzhou, China; ^3^ Department of Medical Imaging, Henan Provincial People’s Hospital, People’s Hospital of Zhengzhou University, Zhengzhou, China; ^4^ Department of Gastroenterology, Henan Provincial People’s Hospital, People’s Hospital of Zhengzhou University, Zhengzhou, Henan, China

**Keywords:** hepatocellular carcinoma, machine learning, radiomics, early recurrence, multidetector computed tomography

## Abstract

**Objective:**

To develop a contrast-enhanced computed tomography (CECT) based radiomics model using machine learning method and assess its ability of preoperative prediction for the early recurrence of hepatocellular carcinoma (HCC).

**Methods:**

A total of 297 patients confirmed with HCC were assigned to the training dataset and test dataset based on the 8:2 ratio, and the follow-up period of the patients was from May 2012 to July 2017. The lesion sites were manually segmented using ITK-SNAP, and the pyradiomics platform was applied to extract radiomic features. We established the machine learning model to predict the early recurrence of HCC. The accuracy, AUC, standard deviation, specificity, and sensitivity were applied to evaluate the model performance.

**Results:**

1,688 features were extracted from the arterial phase and venous phase images, respectively. When arterial phase and venous phase images were employed correlated with clinical factors to train a prediction model, it achieved the best performance (AUC with 95% CI 0.8300(0.7560-0.9040), sensitivity 89.45%, specificity 79.07%, accuracy 82.67%, p value 0.0064).

**Conclusion:**

The CECT-based radiomics may be helpful to non-invasively reveal the potential connection between CECT images and early recurrence of HCC. The combination of radiomics and clinical factors could boost model performance.

## Introduction

HCC is the most common type of liver malignant tumor and ranked third among all cancer-related death worldwide (1, 2). The global prevalence rate of HCC has an upward trend, especially in eastern Asia and sub-Saharan Africa where approximately 80% of HCC cases are located (3). Hepatic resection is considered as the mainstay curative treatment options of HCC with early stage and preserved liver function (2). However, HCC is still a major medical problem worldwide because of its complex pathogenesis and poor prognosis (4). The definition of early recurrence of HCC is the first appearance of new liver tumor lesions or metastasis, or histopathologically confirmed recurrence within 1 year after resection, and early recurrence of HCC accounts for over 70% of HCC recurrence cases (5–7). The 5-year survival rate of HCC is only 12%, and the main reason of its poor prognosis is the high risk of postoperative early recurrence (8). Therefore, the accurate prediction of early recurrence is critical to patient risk stratification, clinical decision-making, optimal surveillance, and survival of HCC.

Traditionally, HCC staging systems such as TNM system and Barcelona Clinic Liver Cancer play a main role in the treatment and prognosis of HCC (9). However, most of the factors used in these systems can only be obtained after surgery, so the current staging systems are inadequate for predicting early recurrence of HCC preoperatively. Medical images of HCC play a key role in the screening, diagnosis, and treatment of HCC. However, the full potential of these medical images has not been harvested because of the subjective visual analysis carried out by radiologists (10, 11). How can we predict the early recurrence of HCC with quantitative image analysis? The solution has important clinical value, as well as precision medicine.

Compared to traditional imaging evaluation, radiomics is an emerging technology with the ability to transform medical images into mineable quantitative features (12, 13). Deep learning has shown promising, powerful, and reliable capabilities to tackle large and highly complex machine learning tasks related to medical images (14–16). Several studies have reported the prediction of early postoperative recurrence of HCC by using MRI (7, 17–19) and CT (6, 20, 21). However, no studies have ever employed a deep learning method to construct the prediction model of early recurrence.

Thus, the main purpose of this study is to develop radiomic models using a deep learning method and assess its efficacy in the preoperative prediction of early HCC recurrence. Meanwhile, the performance of these models was also compared in terms of radiomic features and clinical factors.

## Materials and methods

### Subjects and clinicopathological characteristics

Between February 2012 and August 2016, a consecutive series of patients pathologically confirmed with HCC were retrospectively recruited in our study. The inclusion criteria were listed below: (1) patients who underwent liver resection and pathologically diagnosed as HCC, (2) the liver CECT was done within one week before surgery, (3) CECT images were available and the quality could satisfy the analysis requirement, (4) patients with no other cancers, (5) patients have not received other treatment, such as local ablation and transarterial chemoembolization, before surgery, (6) patients with available follow-up data.

The demographics, laboratory examination data, and imageological examination data (e.g., age, gender, alpha-fetoprotein (AFP), aspartate aminotransferase (AST), lymph node enlargement, hepatocirrhosis, etc.) of the patients were retrieved from electronic health record (EHR). The pathological grades of the patients were obtained from the Pathology Information Management System.

The Shapiro-Wilk test were applied to analyze the continuous variable to determine whether they followed normal distribution. Non-normal distribution Variables were expressed as median (25th, 75th percentile). Categorical variables were expressed as frequency and percentage. The chi-squared test and t-test were applied to evaluate the difference in categorical variables and continuous variables, respectively. P-value less than 0.05 indicates a significant statistical difference in patients’ indicators between the training dataset and the test dataset. Statistical analyses were performed via IBM SPSS Statistics 22.0.

### Follow-up surveillance

The follow-up surveillance in this study consisted of laboratory examinations and physical examinations, including AFP levels, liver function tests, and abdominal ultrasonography. The surveillance was performed within 1 month after resection and then every 3-6 months. The endpoint of this study was early recurrence. Follow-up time ranged from 32 days to 1,907 days, the mean and standard deviation of follow-up time were 456.35 and 404.99, respectively.

### Image acquisition

**Figure 1:**
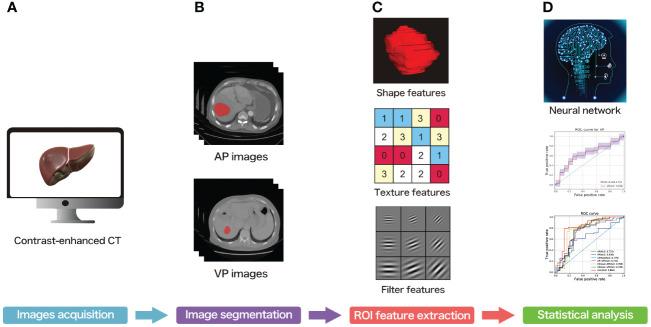
The overall workflow of the present study. **(A)** Contrast-enhanced CT acquisition of all subjects. **(B)** ROI segmentation of each slice. **(C)** ROI feature extraction. **(D)** Statistical analysis.

The overall workflow is shown in **Figure 1**. All patients underwent CECT of the liver using one of the multidetector row CT units (Brilliance 16, Philips or LightSpeed VCT, GE Healthcare, or Discovery CT750 HD, GE Healthcare, or SOMATOM Definition Flash, Siemens) in two modalities, which were called arterial phase (AP) and venous phase (VP). AP and VP were performed after 25 and 60s of delay after intravenous injection of the contrast agent (1.5 ml/kg; Ultravist 370, Bayer HealthCare Pharmaceuticals Inc.), respectively. Sensitive information of the patients was removed, so the images of all patients were anonymized and stored in the DICOM format.

### Tumor segmentation

**Figure 2:**
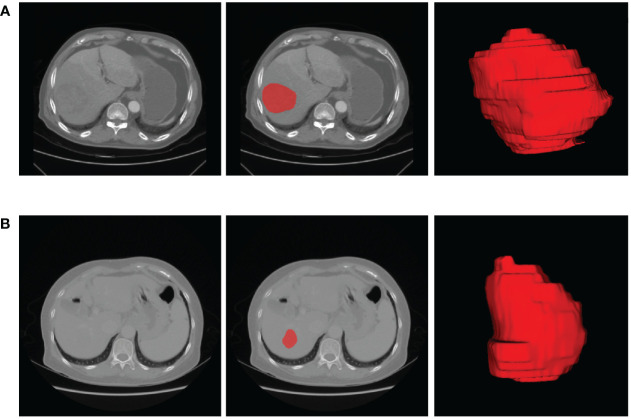
The representative results of ROI segmentation. **(A)** The original AP-CECT, slice segmentation, and 3D effects of the ROI of a 75-year-old male patient with early recurrence. **(B)** The original VP-CECT, slice segmentation, and 3D effects of the ROI of a 65-year-old female patient with non-early recurrence.

The lesion sites were manually segmented by ITK-SNAP Version 3.0. ITK-SNAP is an open-source software that provides the capabilities of semi-automatic segmentation as well as image navigation (22). The process of lesion segmentation was described as follows. First, the AP- and VP-CECT images were imported into the software, respectively. Second, each slice of the images was viewed to get an overview impression of the lesions. Third, under the supervision of a imaging specialist with twenty years of experience, a radiologist with eight years of experience in abdominal imaging manually draw each slice along the boundary of each lesion. The radiologists were blinded to the diagnosis of the patients. The segmentation results are shown in **Figure 2**.

### Feature extraction

The open-source Pyradiomics package Ver. 2.0, which is a reference standard for radiomic analysis, was used to extract features of AP- and VP-CECT images (23). To eliminate the variation caused by different scanners and to improve reproducibility, image preprocessing was implemented utilizing normalization, resampling, and gray-level discretization. More details of image preprocessing were shown in a previous study (24).

Seven categories of features were extracted, and the various features were listed as follows (25). I. First-order features. II. Shape features (3D). III. Gray level co-occurrence matrix (GLCM). IV. Gray level size zone matrix (GLSZM). V. Gray level run length matrix (GLRLM). VI. Neighboring gray tone difference matrix (NGTDM). V. Gray level dependence matrix (GLDM). Besides, 17 filters were employed on the original images to yield derived images. In addition to shape features, other features were calculated on both the derived and the original images.

### Feature preprocessing and data cleaning

In general, machine learning classifiers cannot achieve high performance when the features are on very different scales (26). Feature scaling is one of the most important transformations carried out to provide all features with the same scale. In the present study, normalization was applied and the process was described as below. First, the min value was subtracted. Second, the results of the first step were divided by the max minus the min. Otherwise, the median value and mode of the variables were used to fill the missing data of the continuous and discrete variables, respectively.

### Model establishment and evaluation

To extract high-level semantic features, a deep neural network (DNN) containing input layers, output layers, and several deep stacks of hidden layers was employed. Batch normalization, dropout layer, and exponential linear unit (ELU) were also applied in the present study. ELU can significantly reduce the risk of vanishing gradients at the beginning of training and can map linear features to non-linear features (27). Batch normalization can accelerate the convergence of the model and prevent overfitting (28). Dropout is a regularization technique used to drop out some neurons with a certain probability (29). Keras version 2.1, a simple high-level API for constructing, training and evaluating models, was employed to establish the model. All subjects were randomly allocated to a training set and a test set applying stratified sampling method based on their labels.

**Figure 3:**
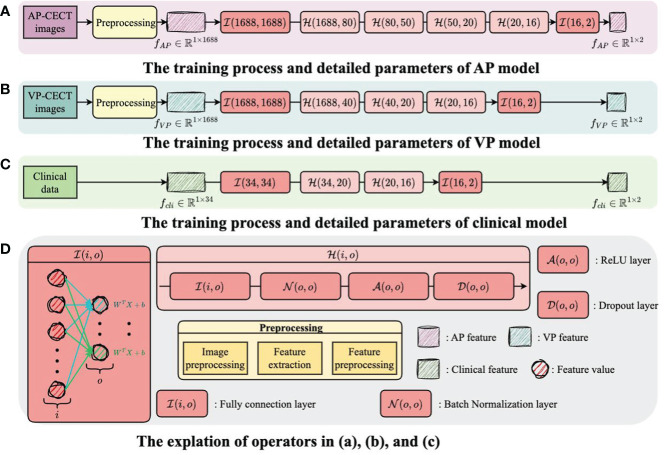
The training flow for single-modality data. **(A)** The training process and detailed parameters of AP model. **(B)** The training process and detailed parameters of VP model. **(C)** The training process and detailed parameters of clinical model. **(D)** The explantion of operators in **(A-C)**.

**Figure 4:**
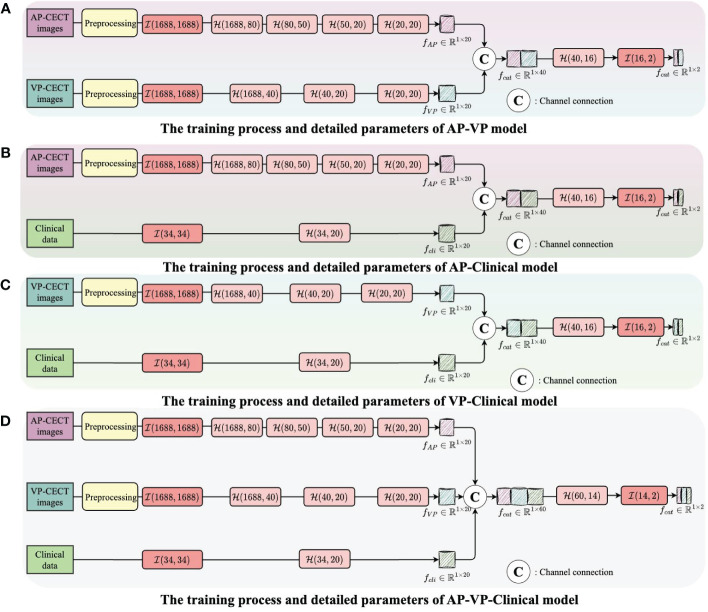
The integration of multi-modality data to enhance the training outcome. **(A)** The training process and detailed parameters of AP-VP model. **(B)** The training process and detailed parameters of AP-Clinical model. **(C)** The training process and detailed parameters of VP-Clinical model. **(D)** The training process and detailed parameters of AP-VP-Clinical model.

The structure and training process of the model are illustrated in **Figure 3** and **Figure 4**, with **Figure 3** showing the training flow for single-modality data and **Figure 4** demonstrating the integration of multi-modality data to enhance the training outcome. For the AP model, we designed a deep network structure to extract high-frequency features from the images, which are crucial for distinguishing different types of early HCC recurrence. These high-frequency features represent subtle variations in the images that may indicate early signs of cancer recurrence. The VP model employs a shallower network to capture local and detailed information, thereby increasing the model’s sensitivity to classification. For the clinical data model, given the lower dimensionality of the information provided, we opted for a simplified network structure focused on extracting clinical indicators critical for disease classification.

Each model’s input layer utilizes a fully connected layer, serving as a hub to collate and preprocess data for subsequent layers. Subsequent nonlinear modules transform the raw data into forms more meaningful for model learning. Batch normalization layers are used to stabilize the training process, while Dropout layers prevent overfitting to specific patterns in the training data, enhancing the model’s ability to generalize.

The process of integrating multi-modality data can be likened to the collective intelligence of an interdisciplinary team collaboration. In the VP-AP model, image data from each modality is processed separately to extract high-frequency features, which are then combined in the feature space, promoting the sharing of knowledge between different modalities. Through this fusion strategy, our model can integrate feature knowledge across modalities and assess the importance of these features for classification, focusing on those that contribute most to the decision-making process. All these efforts are directed towards improving the accuracy of predicting early HCC recurrence, thereby providing stronger support for clinical decision-making and assisting physicians in more accurately assessing patient risk before surgery. The code and data were uploaded to github (https://github.com/luojiadream/Early-Recurrence-of-HCC).

The performance of the deep learning model was evaluated using both the training dataset and the test dataset based on AUC and its 95% confidence intervals (CIs), sensitivity, specificity, and accuracy. Delong’s test was employed to calculate the p-value, which was then used to evaluate the degree of fitting of the models.

## Results

### Clinicopathological characteristics of the patients

According to the inclusion criteria, 297 patients were included in this study, and they were assigned to a training set (n = 237) and a test set (n = 60). This bifurcation was meticulously conducted to furnish the model with a comprehensive learning set (training set) and a discrete validation cohort (test set) to objectively evaluate the predictive prowess of the model. The training set, the primary educational substrate for the model, includes 213 male patients, representing 89.9% of the assemblage. The median and standard deviation of age in the training set and the test set were 53.3 ± 10.2 and 53.2 ± 9.1, respectively. There were 219 and 6 patients with Hepatitis B and Hepatitis C in the training set, respectively. There were 157 and 38 patients in the training set and test set, respectively, whose AFP was out of limits. 23, 201, 15, 186, and 200 patients in the training dataset showed CT features of lymph node enlargement, single tumor, absent liver cirrhosis, incomplete capsule appearance, and smooth tumor margin, respectively.

The distribution of these patients into training and test sets was executed with precision, ensuring the model is calibrated on a substantive dataset, while validation is performed on a distinct set to affirm the model’s accuracy and reliability in novel clinical scenarios. There was no significant difference in the clinical indicators between the training set and the test set (all p > 0.05). The detailed clinical indicators of the patients are listed in **Table 1**.

**Table 1:** The detailed clinicopathological characteristics of the subjects in the training dataset and the test dataset.

Characteristic	Training dataset (n=237)	Test dataset (n=60)	P-value
Patient demographics			
**Gender ǂ**			0.727
Male	89.9 (213)	88.3 (53)	
Female	10.1 (24)	11.7 (7)	
Age (y)*	53.3±10.2	53.2±9.1	0.942
**Liver disease ǂ**			0.925
Hepatitis B virus infection	92.4 (219)	93.3 (56)	
Hepatitis C virus infection	2.5 (6)	1.7 (1)	
Others	5.1 (12)	5.0 (3)	
Laboratory examination			
AFP **ǂ**	66.2 (157)	63.3 (38)	0.671
GGT (U/L)*	67.0 (35.3, 119.0)	58.5 (34.5, 117.8)	0.854
AST (U/L)*	40.5 (28.0, 57.0)	41.0 (29.5, 65.3)	0.593
ALT(U/L)*	42.5 (29.0, 64.8)	44.0 (29.3, 73.5)	0.463
**CT features**			
Tumor diameter(mm)*	53.3 (33.7, 82.8)	46.8 (34.9, 76.1)	0.520
Lymph node enlargement **ǂ**	9.7 (23)	8.30 (5)	0.745
Tumor number **ǂ**			0.551
Solitary	84.8 (201)	81.7 (49)	
Multiple	15.2 (36)	18.3 (11)	
Liver cirrhosis **ǂ**			0.999
Absent	6.3 (15)	6.7 (4)	
Present	93.7 (222)	93.3 (56)	
Capsule appearance **ǂ**			0.394
Incomplete	78.5 (186)	73.3 (44)	
Complete	21.5 (51)	26.7 (16)	
Tumor margin **ǂ**			0.907
Smooth	84.4 (200)	85.0 (51)	
Nonsmooth	15.6 (37)	15.0 (9)	
Histologic characteristics ǂ			
Edmondson grade			0.434
I-II	61.2 (145)	66.7 (40)	
III-IV	38.8 (92)	33.3 (20)	
**Number of early recurrence**	66.2 (157)	76.7 (46)	0.121

P-value < 0.05 indicates a significant difference in patients’ characteristics between the training dataset and test dataset.

*Continuous variables , data are medians with interquartile range in parentheses.

**ǂ**Categorical variables, data are percentages with numbers of patients.

AFP, Alpha-fetoprotein; ALT, Alanine aminotransferase; AST, Aspartate aminotransferase; GGT, Gamma-glutamyl transpeptidase.

### Feature extraction

1,688 radiomic features were extracted from AP- and VP-CECT, respectively. The number of extracted features of first-order statistics, shape-based, GLCM, GLDM, GLRLM, GLSZM, and NGTDM was 18, 14, 24, 14, 16, 16, and 5, respectively. The extracted features are listed in **Supplementary Appendix**.

### The performance of the prediction model

**Figure 5:**
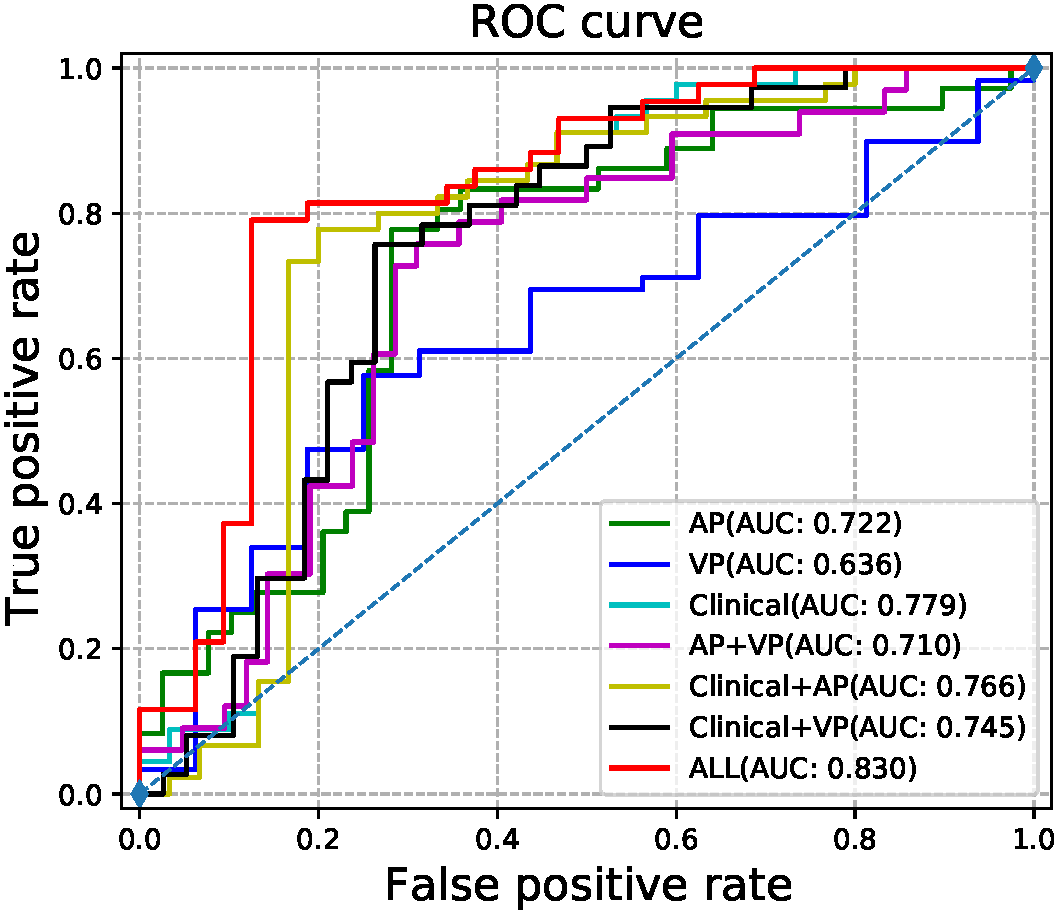
The ROC curves of the models.

The radiomic signatures of AP-CECT images and clinical factors showed high-performance in differentiating early recurrence HCC from non-early recurrence HCC, and the AUC was 0.7215 (95% CI, 0.6490-0.7940) and 0.7793 (95% CI, 0.7030–0.8560), respectively. The VP-CECT images performed worst, and the AUC was 0.6238 (95% CI, 0.5900-0.6580). When arterial phase and venous phase images were employed correlated with clinical factors, the model performed best with an AUC of 0.8300(95% CI, 0.7560-0.9040). All p-values were less than 0.0125, indicating a significant difference in the classification of early recurrence. The ROC of the seven established models is displayed in **Figure 5**, and the detailed results are listed in **Table 2**.

**Table 2:** The detailed prediction performance of the radiomic signature, the clinical factors, and the combined model.

Methods	Training dataset(n=237)					Test dataset(n=60)				
	Sensitivity (%)	Specificity (%)	Accuracy (%)	AUC (95% CI)	P Value	Sensitivity (%)	Specificity (%)	Accuracy (%)	AUC (95% CI)	P Value
AP	68.00	84.30	77.74	0.7623(0.7270-0.7980)	0.0028	71.05	75.00	73.33	0.7215(0.6490-0.7940)	0.0059
VP	80.67	57.89	61.13	0.6238(0.5900-0.6580)	0.0045	80.67	57.63	61.13	0.6238(0.5900-0.6580)	0.0069
Clinical	79.33	80.41	80.01	0.7902(0.7560-0.8250)	0.0075	86.84	73.33	77.33	0.7793(0.7030-0.8560)	0.0098
AP+VP	57.33	75.44	69.44	0.6592(0.6240-0.6940)	0.0037	65.79	75.76	72.00	0.7100(0.6360-0.7840)	0.0064
AP+Clinical	84.67	67.20	71.76	0.7050(0.6720-0.7390)	0.0030	92.10	72.91	78.67	0.8125(0.7390-0.8860)	0.0060
VP+Clinical	73.33	83.33	79.40	0.7693(0.7350-0.8040)	0.0046	76.32	76.32	76.00	0.7333(0.6560-0.8150)	0.0069
AP+VP+Clinical	85.33	80.00	82.06	0.7871(0.7530-0.8210)	0.0037	89.45	79.07	82.67	0.8300(0.7560-0.9040)	0.0064

P-value was adjusted to 0.0125 according to Bonferroni correction. P-value < 0.125 indicates a significant difference in the discrimination of early recurrence HCC and non-early recurrence HCC.

AP, Arterial phase; VP, Venous phase.

## Discussion

Radiomics is the application of new technology which aims to reveal the potential relationship between medical images and phenotypic characteristics of tumor cells (24). It has been provided new perspectives in HCC related to prediction of histology, response to treatment, genetic signature, recurrence, and survival. Early recurrence of HCC accounts for over 70% of HCC recurrence cases, it is the main reason for the poor prognosis of HCC. Clinically, preoperative prediction of early recurrence of HCC is critical to patient risk stratification, optimal clinical decision-making, and subsequent follow-up. Current staging systems are inadequate for predicting early recurrence of HCC preoperatively. On the contrary, CECT imaging could systematically explore cell proliferation, liver function, and prognosis of hepatopathy (30).

Our study aimed to develop a CECT-based radiomics model by using a deep learning method to improve the accuracy of prediction. In this study, 1,688 radiomic features were extracted from AP- and VP-CECT images, respectively. Then, seven models were constructed based on various combinations of radiomic features and clinical factors. The study showed that AP, VP and clinical factors could be used to successfully predict early recurrence of HCC separately, all achieving p-values were less than 0.0125 in the training set and the test set. The combination of radiomic features and clinical factors could boost model performance, and this conclusion was consistent with that of previous studies (6, 7, 21). The performance of AP was better than VP because the arterial blood supply of HCC was abundant, and the intensity of AP signals was higher than that of VP in the tumor parenchyma (31).

The prediction of early recurrence of HCC has become a hot research field, however, deep learning method has never been employed in those studies. Previous studies have reported that radiomics may be helpful to the preoperative prediction of early recurrence of HCC, we selected 15 representative papers and compared them with our study in terms of imaging modality, number of included patients, modeling methods, etc. From the perspective of image modality, CT(n=6) (6, 20, 21, 32–34) has higher specificity than MRI (n=9) (7, 35–42). In our study, we used CECT which hold fixed and uniform parameters than MRI to predict early recurrence. In addition, compared with CT, MRI image acquisition technology is more complex and more sensitive to alpha shadow and image quality inhomogeneity in diagnosing HCC. Therefore, this may lead to better prediction results of CT radiomics than MRI radiomics. From the perspective of the number of included patients, only four studies had more patients than our study (6, 35, 36, 38). In machine learning-based radiomics research, the number of included patients is the basis of the research. The larger the number of patients, the better of the generalization and the higher of the feasibility. From the perspective of the modeling methods, among the 14 studies participating in the comparison, modeling methods are logistic regression or lasso regression, the number is 6 and 8, respectively. In our study, we applied a relatively novel deep learning method and achieved better model prediction effects and generalization. In 2007, a paper published by Segal, E. et al. in Nature Biotechnology showed that medical image could reconstructed 78% of the global gene expression profiles, revealing cell proliferation, liver synthetic function, and patient prognosis (30). Subsequently, more and more scholars have affirmed the importance of medical imaging in cancer research. We also believe that clinical characteristics play an important role in predicting early recurrence of HCC (43). However, as the amount of data increases significantly, simply utilizing clinical features cannot bring the same level of performance improvement in deep learning-based models. In this study, although the performance of the combination model is not much better than that of the clinical feature model, the performance of our combination model based on deep learning will become more and more prominent as the amount of data increases. In view of the important role of multimodal medical imaging in radiomics research and the excellent performance of deep learning models in terms of model generalization and data fitting performance, etc. Therefore, it is valuable to develop a multi-modal combination model based on deep learning to predict early recurrence of HCC.

In summary, compared with other studies, our contributions are mainly reflected in two aspects. First, from the methodological perspective, we applied deep learning-based radiomics methods to reveal the potential relationship between tumor imaging features and early recurrence, improving the prediction performance. Second, the features extracted from AP images were more reliable for predicting early recurrence of HCC, the findings are consistent with the blood supply characteristics of HCC.

Although our study has many innovations, it also has certain limitations. First, the study is a single center research. Thus, the reproducibility of the study needs to be verified. Second, the sample size of this study was relatively small. Third, only operable cases were included, excluding unresectable disease likely representing later-stage cancers, so the patient cohort exhibits selection bias.

In summary, the deep learning-based CECT radiomic analysis could improve the prediction accuracy, and the combination of radiomic and clinical factors could boost the prediction performance.

## Data availability statement

The original contributions presented in the study are included in the article/**Supplementary Material**. Further inquiries can be directed to the corresponding authors.

## Author contributions

BM: Methodology, Writing – original draft. YR: Data curation, Investigation, Writing – review & editing. XY: Software, Validation, Writing – review & editing. XL: Supervision, Writing – review & editing. XD: Funding acquisition, Supervision, Validation, Writing – review & editing.
